# Crosstalk among N6-methyladenosine modification and RNAs in central nervous system injuries

**DOI:** 10.3389/fncel.2022.1013450

**Published:** 2022-09-29

**Authors:** Mi Tian, Lei Mao, Li Zhang

**Affiliations:** ^1^Department of Anesthesiology, Affiliated Zhongda Hospital of Southeast University, Nanjing, Jiangsu, China; ^2^Department of Neurosurgery, Jinling Hospital, School of Medicine, Nanjing University, Nanjing, Jiangsu, China

**Keywords:** central nervous system injuries, m6A modification, neurological impairment, apoptosis, inflammation, downstream molecules

## Abstract

Central nervous system (CNS) injuries, including traumatic brain injury (TBI), intracerebral hemorrhage (ICH) and ischemic stroke, are the most common cause of death and disability around the world. As the most common modification on ribonucleic acids (RNAs), N6-methyladenosine (m6A) modification has recently attracted great attentions due to its functions in determining the fate of RNAs through changes in splicing, translation, degradation and stability. A large number of studies have suggested that m6A modification played an important role in brain development and involved in many neurological disorders, particularly in CNS injuries. It has been proposed that m6A modification could improve neurological impairment, inhibit apoptosis, suppress inflammation, reduce pyroptosis and attenuate ferroptosis in CNS injuries *via* different molecules including phosphatase and tensin homolog (PTEN), NLR family pyrin domain containing 3 (NLRP3), B-cell lymphoma 2 (Bcl-2), glutathione peroxidase 4 (GPX4), and long non-coding RNA (lncRNA). Therefore, m6A modification showed great promise as potential targets in CNS injuries. In this article, we present a review highlighting the role of m6A modification in CNS injuries. Hence, on the basis of these properties and effects, m6A modification may be developed as therapeutic agents for CNS injury patients.

## Introduction

Central nervous system (CNS) injuries and their potential long-term consequences are of major concern for public health. High rates of morbidity and mortality making them a global health challenge (Hornby et al., 2020). CNS is highly sensitive to external mechanical damage, such as traumatic brain injury (TBI), spinal cord injury (SCI), subarachnoid hemorrhage (SAH), and stroke, presenting a limited capacity for regeneration due to its inability to restore either damaged neurons or synaptic network (Zhang and Wang, 2019). Although some of the pathological processes of CNS injuries such as blood brain barrier (BBB) disruption, inflammation and oxidative stress have been elucidated, the detailed mechanisms driving these processes are poorly understood (Devanney et al., 2020). Despite the progress has been made in the prevention and treatment of CNS injuries in the past, patients suffering from CNS injuries usually end up with poor prognosis (Liddelow and Barres, 2017). Therefore, it is urgently needed to find optimal therapies and improve patients’ long-term neurological functioning after CNS injuries.

N6-methyladenosine (m6A) was firstly reported in 1974 (Reichel et al., 2019). It is evolutionarily conserved, ranging from yeasts, plants, insects to mammals (Malovic et al., 2021). M6A modification is one of the most common epigenetic modifications for eukaryotic ribonucleic acids (RNAs), not only in messenger RNAs (mRNAs) but also in a variety of non-coding RNAs (ncRNAs) such as long non-coding RNAs (lncRNAs) and circular RNAs (circRNAs) (Zhou L. et al., 2022). The modification process of m6A is regulated by methyltransferases (writers), demethylases (erasers), and RNA binding proteins (RBPs; readers) (Jang et al., 2022). Specifically, the sixth nitrogen atom on the RNA molecule is methylated by the catalysis of methyltransferase, the methylation site is then identified by RBPs and participates in the pathophysiological processes *via* mediating RNA splicing, transcription, translation and decay (Chen D. et al., 2022; Jang et al., 2022). However, the methylation sites can be demethylated under the effect of demethylase, indicating a dynamic and reversible process (Luo et al., 2022). Recently, the regulation role of m6A modification in CNS has gradually been explained. M6A is enriched in RNAs of neurogenesis, cell cycle and neuron differentiation (Chokkalla et al., 2020; Pan et al., 2021). The dysregulation of writer, eraser, and reader proteins of m6A modification is associated with the occurrence and conversion of neurological diseases including CNS injuries (Wang et al., 2021; Zhai et al., 2022).

In the present study, we provide an overview of m6A modification in CNS injuries and the associated molecular mechanisms. This review describes (1) Abnormal expression of m6A modification proteins in CNS injuries; (2) Detecting methods of m6A modification; (3) Function of m6A modification in CNS injuries; (4) Downstream molecules of m6A modification in CNS; (5) Crosstalk between m6A modification and RNAs in CNS injuries, and (6) Possible research directions of m6A modification in CNS injuries.

## Abnormal expression of N6-methyladenosine modification proteins in central nervous system injuries: Writers, erasers and readers

**FIGURE 1:**
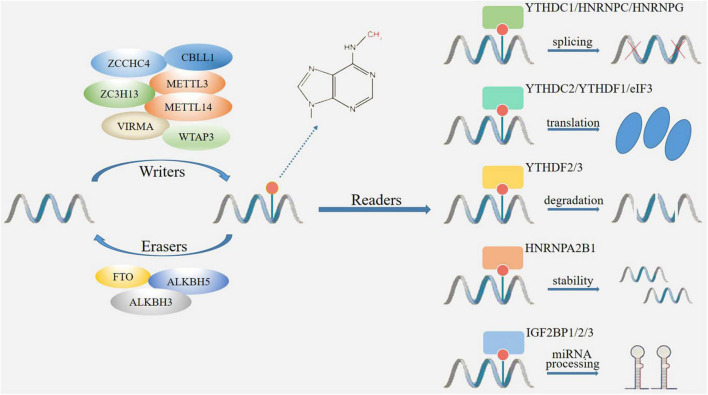
The mechanism of m6A modification. M6A modification is catalyzed by methyltransferase and demethylase acting as writers (METTL3, METTL14, WTAP3, VIRMA, ZC3H13, ZCCHC4, CBLL1) and erasers (FTO, ALKBH3, ALKBH5) to add and remove m6A. The m6A binding proteins (readers) include YTH and IGF2BP family proteins, which determine the fate of RNAs through changes in splicing, translation, degradation, stability, and miRNA processing.

The m6A modification-related proteins can act as writers or erasers to add or remove m6A, respectively, that means, these proteins determine whether m6A is methylated or demethylated. While, RBPs called readers recognize m6A sites to interact with RNA (Zhang F. et al., 2022). At present, it has been found that there was abnormal expression of m6A modification regulatory factors in CNS injuries, which may explain their pathological mechanisms (Figure 1).

### Writers

Writers, also known as methyltransferase complexes (MTCs), are a class of functional proteins that promote the methylation modification of adenine base sites (Cai et al., 2022). The core proteins of m6A methyltransferase include methyltransferase-like protein 3 (METTL3), METTL14, Wilms tumor 1-associated protein (WTAP), Vir-like m6A methyltransferase-associated (VIRMA) and zinc finger CCCH-type containing 13 (ZC3H13) (Yang X. et al., 2022). METTL3 is the critical component protein in m6A MTCs. METTL3 has its own catalytic ability, which can bind with methyl alone to catalyze the formation of m6A, and promote protein translation in the cytoplasm (Wang and Zhou, 2022). Although METTL14 has no catalytic function and cannot promote the formation of m6A independently, it can form a heterodimer with METTL3 and recognizing the m6A specific sequence (Zhang and Liu, 2022). Therefore, these MTCs are not independent in organisms, they can form complexes to co-perform catalytic functions. WTAP owns the functions of stabilizing heterodimers, locating nuclear spots, facilitating RNA degradation and regulating cell differentiation. WTAP can promote m6A modification by guiding METTL3-14 heterodimer localization to nuclear spots (Akcaoz and Akgul, 2022). VIRMA, also known as KIAA1429, regulates m6A mRNA level by recruiting methyltransferase METTL3/METTL14/WTAP complex through its N-terminal (Sommerkamp et al., 2022). Under the recruitment of VIRMA, METTL3, and METTL14 form a heterodimer. Then WTAP stabilizes the heterodimer and forms the m6A MTC in the nucleus. The m6A MTC promotes the transfer of methyl from the donor substrate S-adenosylmethionine, and combines with the nitrogen-containing base at the sixth position of adenylate to form m6A (Cayir, 2022). ZC3H13 can combine with WTAP to promote MTC deposition in nuclear and enhance m6A modification (Song et al., 2022). Recently, some novel methyltransferases have been recognized. For example, METTL5 has been shown to catalyze m6A installation by forming a METTL5-TRMT112 heterodimeric complex to increase metabolic stability, suggesting a novel RNA-binding pattern different from the METTL3-14 heterodimer (Oerum et al., 2021).

It has been indicated that CNS injuries could change the expression of writers. For example, in a mouse TBI model, genome-wide profiling of m6A-tagged transcripts was conducted by m6A-modified RNA immunoprecipitation sequencing (m6A-RIP-seq) and RNA sequencing (RNA-seq). The results showed that METTL3 was downregulated after TBI. In addition, 922 m6A peaks were differentially expressed as determined by m6A-RIP-seq, with 370 upregulated and 552 downregulated (Wang et al., 2019). Furthermore, in a rat TBI model, Yu et al. conducted a genome-wide profiling of mRNA m6A methylation in rat cortex *via* methylated RIP sequencing (MeRIP-Seq). They found that after TBI, the expressions of METTL14 were significantly down-regulated in rat cerebral cortex (Yu et al., 2020).

### Erasers

Erasers, also known as demethylase complexes, demethylate m6A modification *via* demethylases such as fat mass and obesity associated protein (FTO) and α-ketoglutarate-dependent dioxygenase alk B homolog 5 (ALKBH5), thereby maintaining the dynamic and reversible of m6A modification (Li Y. et al., 2022). FTO is the first discovered demethylase that belongs to the ALKB family. It is an oxygenase dependent on Fe^2+^ and 2-oxoglutarate, which can catalyze the demethylation of nucleotides (Li L. et al., 2022). ALKBH5 is found after FTO, it is Fe^2+^ and α- Ketoglutarate (α-Kg) dependent non-heme oxygenase (Imanishi, 2022). Although both FTO and ALKBH5 are demethylases, they are differentially distribution in mammalian organs and have different substrate preferences. Firstly, FTO is enriched in brain, especially in neurons, and plays an important regulatory role in CNS. While ALKBH5 is highly expressed in testes and is essential for spermatogenesis. Secondly, although FTO and ALKBH5 are both demethylases of m6A, the main substrate of FTO is N6-hydroxymethyladenosine (hm6A) and N6-formyladenosine (fm6A) but the main substrate of ALKBH5 is original adenylate. Thirdly, FTO-mediated demethylation process is gradually completed. M6A is firstly converted to hm6A, then transformed to fm6A, and finally reduced to original adenylate. However, ALKBH5 can directly reduce m6A to original adenylate (Kumari et al., 2022; Song et al., 2022). The difference between FTO and ALKBH5 in the metabolic mechanism of m6A results in different biological functions (Chen J. et al., 2022). Recently, ALKBH3 has been suggested to be a novel demethylase protein which mediates the demethylation of m6A, m1A and 3-methylcytidine (m3C) on tRNA (Liu et al., 2021).

The expression of erasers in CNS injuries has also been analyzed. In a rat stroke model, the protein levels of demethylases FTO and ALKBH5 were changed, both FTO and ALKBH5 co-regulated m6A demethylation, which played a crucial role in cerebral ischemia/reperfusion (I/R) injury (Xu K. et al., 2020).

### Readers

Readers, also named as m6A methylation RBPs, can bind to specific m6A methylation sites of RNAs and determine the fates of m6A-methylated RNAs (Zhou H. et al., 2022). By recruiting specific methylation RBPs on RNAs, m6A interacts with its substrate to regulate different molecular effects, including RNA processing, mRNA enucleation, translation and shearing (Zhou W. et al., 2022). Currently, there are three RBPs that have been widely studied, including YT521-B homology (YTH) domain family proteins, insulin-like growth factor 2 mRNA-binding proteins (IGF2BPs) and heterogeneous nuclear ribonucleoproteins (HNRNP) family proteins (Liu C. et al., 2022). The YTH domain family proteins are important m6A methylation RBPs, which can bind to m6A and affect the outcome of m6A-methylated RNAs (Yan et al., 2022). YTH proteins mainly includes YTHDFs and YTHDCs subtypes. YTHDFs subtypes locate in the cytoplasm, among them YTHDF1 promotes mRNA translation, YTHDF2 induces RNA degradation and YTHDF assists YTHDF1/2 to exhibit functions (Liao J. et al., 2022). YTHDCs subtypes locate in the nucleus, among them YTHDC1 affects RNA splicing, enucleation and gene silencing, YTHDC2 suppresses RNA stability and promotes RNA translation (Han et al., 2021). In contrast to the YTH family proteins, IGF2BP proteins, including IGF2BP1/2/3, modulate gene expression output by increasing the stability and translation efficiency of m6A-modified RNAs (Ramesh-Kumar and Guil, 2022). HNRNP family proteins, such as HNRNPC, are located in the nucleus. HNRNPC can recognize and bind to m6A-modified regions in RNAs, thus affecting the abundance and alternative splicing of target RNAs (Huang et al., 2021).

The expression of readers was also changed after CNS injuries. In a rat cerebral ischemia model, YTHDC1 was up-regulated in the early phase of ischemic stroke. Knockdown of YTHDC1 exacerbated ischemic brain injury and overexpression of YTHDC1 protected rats against brain injury (Zhang Z. et al., 2020).

## Detecting methods of N6-methyladenosine modification

In the past decades, due to the limitation in technical means, the detection of m6A and the identification of m6A at the single-base level had been progressing slowly (Shulman and Stern-Ginossar, 2020; Wang Y. N. et al., 2020). In recent years, with the continuous exploration and research on m6A, a number of methods have been developed to detect m6A modification, which further promoted m6A research.

Current methods for m6A detection are based on RNA chemistry or immunoprecipitation to assess an overall transcriptome-wide m6A level and high-throughput sequencing to evaluate the precise location of m6A sites. Dot blot technology (Nagarajan et al., 2019), chemical proteomics approach (Arguello et al., 2017) and high-performance liquid chromatography-mass spectrometry (HPLC-MS) method (Zhang Y. et al., 2020) are common approaches to observe overall m6A changes in transcriptome-wide level. However, they do achieve the quantitation or semi-quantitation, but fail to precisely locate the m6A sites (Zhang Y. et al., 2020). To date, methylated RNA immunoprecipitation (MeRIP)-seq (Meyer et al., 2012) and m6A-seq (Dominissini et al., 2012) are the most used approaches for the detection of m6A sites. These new methods are based on the high specificity of antibodies against m6A, and its combination with high-throughput sequencing makes it possible to describe the specific map of m6A modification in the mammalian transcriptome. The first step is to fragment the RNA, followed by the use of immuno-magnetic beads with m6A antibody to enrich the m6A-methylated RNA fragments and the purification of the enriched RNA fragments to construct a high-throughput sequencing library by performing on-machine sequencing. In addition, a common transcriptome library needs to be constructed separately as a control. Finally, the two sequencing libraries are put together for bioinformatics analyses, and the region with a higher degree of m6A methylation is obtained, which is also called m6A peak (Dominissini et al., 2012; Meyer et al., 2012). However, the approaches can only detect m6A peaks but not acquire the precise position of m6A residues.

To overcome the shortcoming, a novel method called m6A-specific ultraviolet crosslinking immunoprecipitation sequencing technology (miCLIP) was developed to achieve m6A sequencing at single-base resolution (Linder et al., 2015). MiCLIP is the combination of ultraviolet radiation-induced crosslinking coupled with immunoprecipitation sequencing (CLIP-seq) and MeRIP-seq. CLIP-seq can reveal interactions between RNA and RNA-binding proteins at the genome-wide level (Stojkovic et al., 2021). Therefore, this combination can provide m6A information beyond the conserved RRACH sequence, which compensates for the deficiency of MeRIP-seq (Linder et al., 2015; Wang Y. et al., 2020). In addition, several other approaches such as m6A level and isoform-characterization sequencing (m6A-LAIC-seq) (Molinie et al., 2016), site-specific cleavage and radioactive labeling followed by ligation-assisted extraction and thin-layer chromatography (SCARLET) (Liu et al., 2013) and MAZTER-seq (Garcia-Campos et al., 2019) were newly developed to achieve more precise location even at single-nucleotide resolution.

## Functions of N6-methyladenosine modification in central nervous system injuries

Regulation of m6A modification was firstly reported to exhibit neuroprotection on CNS injuries in Wang et al. (2019). Subsequently, a growing number of studies have demonstrated that modulation of m6A modification could provide neuroprotective effects in CNS injuries. The neuroprotection of m6A modification was reportedly attributed to its effects on improvement of neurological impairment, inhibition of inflammation, suppression of apoptosis, reduction of pyroptosis and regulation of ferroptosis (Table 1).

**TABLE 1:** Functions of m6A modification in CNS injuries.

Mechanisms	Factors	Associated molecules
Improve neurological impairment	Reduce neuronal loss in cortex and hippocampus	/
Suppress apoptosis	Reduce chromosomal DNA fragmentation and formation of apoptotic bodies	Bcl-2, caspase-3
Inhibit inflammation	Decrease inflammatory factors and attenuate inflammatory response	NF-κB, TNF-α, IL-1β, *IL-10*
Reduce pyroptosis	Inhibit the formation of inflammasomes	NLRP3, GSDMD-N, caspase-1
Attenuate ferroptosis	Suppress iron-mediated lipid free radical formation and accumulation	GPX4, GSH

CNS, central nervous system; DNA, deoxyribonucleic acid; Bcl-2, B-cell lymphoma-2; NF-κB, nuclear factor kappa-light-chain-enhancer of activated B cells; TNF-α, tumor necrosis factor-α; IL-1β, interleukin-1β; IL-10, interleukin-10; NLRP3, NLR family pyrin domain containing 3; GSDMD-N, N-terminal fragment of gasdermin D; GPX4, glutathione peroxidase 4; GSH: glutathione.

### Neurological impairment

Neurological impairment covers a wide range of illnesses and injuries. Depending on the severity and type of impairment, people can be more or less affected (Kitzmuller et al., 2019). Neurological impairment may occur in CNS injuries, which is characterized by problems in sensory-motor, cognitive and psychological functions, thereby affecting the quality and meaningfulness of life (Benedictus et al., 2010; Hilton et al., 2018). Neurological impairment is a main determinant of functional disability, people suffering from neurological impairment may be unable to use both arms and legs sufficiently, leaving them highly dependent on others (Odgaard et al., 2017).

The effects of m6A modification on neurological impairment after CNS injuries have been studied. In a rat TBI model, a genome-wide profiling of mRNA m6A methylation in rat cortex was conducted *via* MeRIP-Seq. The analysis of both m6A peaks and mRNA expression revealed that there were 175 mRNAs significantly altered methylation and expression after TBI. Of these mRNAs, the expression of FTO was significantly down-regulated. Moreover, FTO inhibitor FB23-2 increased the modified neurological severity score (mNSS) score of rats after TBI, suggesting that inhibition of FTO exacerbated damage to neurological function caused by TBI (Yu et al., 2020). Furthermore, in a rat ischemic stroke model, overexpression of m6A reader YTHDC1 alleviated brain neurological deficits as measured by NSS through promoting phosphatase and tensin homolog (PTEN) mRNA degradation to increase protein kinase B (AKT) phosphorylation (Zhang Z. et al., 2020).

The precise mechanisms underlying how m6A modification regulated neurological impairment were unclear. It has been revealed that neurological impairment involved selective neuronal loss in the hippocampus and cortex (Wang Z. et al., 2014). Therefore, m6A modification may improve neurological impairment by intervene with these pathological processes.

### Inflammation

Inflammation is one of the major determinants of secondary brain damage after CNS injuries (Shang et al., 2019). In normal conditions, inflammation is a vital physiological immune response against noxious stimuli (such as injury or infection) and defends the host against pathogenic threats (Yang and Zhou, 2019). However, in respond to CNS injuries, excessive inflammation may provoke substantial detrimental effects (Shi et al., 2019).

Numerous studies have proposed that m6A modification exerted a central effect in CNS injuries-induced inflammation. Wang B. et al. (2022) demonstrated that in a sepsis brain injury (SBI) model, lipopolysaccharide (LPS) increased the levels of tumor necrosis factor-α (TNF-α), interleukin-1β (IL-1β), IL-6 and decreased the levels of IL-10 in 1321N1 cells, indicating that LPS treatment induced inflammation in SBI. Treatment of emodin suppressed inflammation as evidenced by decreased levels of TNF-α, IL-1β, IL-6, and increased levels of IL-10 in LPS-treated 1321N1 cells. Moreover, emodin up-regulated m6A levels by activation of METTL3, knockdown of METTL3 reversed the effects of emodin on inflammation in SBI. These data demonstrated that emodin inhibited the inflammation of LPS-treated 1321N1 cells by regulation of METTL3 (Wang B. et al., 2022).

The underlying mechanisms of m6A modification-mediated inflammation are immensely complicated. Studies have indicated that the NF-κB signaling pathway might be the key target. It has been shown that METTL14 played a vital role in macrophage inflammation in atherosclerosis *via* the NF-κB/IL-6 signaling pathway (Zheng et al., 2022). Furthermore, METTL3 participated in sympathetic neural remodeling post-myocardial infarction (MI) *via* the NF-κB pathway and reactive oxygen species (ROS) production. Knockdown of METTL3 inhibited the activation of NF-κB pathway by suppressing the binding of METTL3 to tumor necrosis factor receptor-associated factor 6 (TRAF6), reducing the m6 A level of TRAF6 mRNA and TRAF6 expression, thus inhibiting ROS production and inflammatory responses. These data suggested that METTL3 played an important role in ROS production and inflammatory responses by regulating the TRAF6/NF-κB axis (Qi et al., 2022). Thus, m6A modification may also regulate inflammation *via* NF-κB in CNS injuries, further studies are needed to confirm it.

### Apoptosis

Apoptosis is a very tightly programmed cell death (PCD) occurring regularly to eliminate unnecessary and unwanted cells as well as to maintain a homeostatic balance between cell survival and cell death (Quillinan et al., 2016). Apoptosis is critical to animals especially long-lived mammals that must integrate multiple physiological and pathological death signals (Rodriguez et al., 2021). It has been shown that insufficient apoptosis can trigger cancer or autoimmunity, while excessive activation of apoptosis could contribute to abnormal cell death (Wang X. X. et al., 2020).

The functions of m6A modification in apoptosis have been explored. The results obtained by Xu S. et al. (2020) demonstrated that in an *in vitro* ischemic stroke model, oxygen and glucose deprivation/reoxygenation (OGD/R) treatment in SH-SY5Y cells induced apoptosis as evidenced by cleavage of caspase-3-poly (ADP-ribose) polymerase (PARP) and increase of Annexin V-positive staining. Additional experimental results showed that OGD/R induced mitochondrial depolarization and caused accumulation of JC-1 green monomers fluorescence. In addition, MeRIP-quantitative real-time polymerase chain reaction (qPCR) results showed that OGD/R induced METTL3-dependent lncRNA D63785 m6A methylation, knockdown of METTL3 caused accumulation of lncRNA D63785, thus suppressing neuronal cell death and apoptosis (Xu S. et al., 2020). In another study conducted by Xu K. et al. (2020) they found that the RNA m6A levels increased consecutive to the decrease of FTO expression in rats after middle cerebral artery occlusion (MCAO) and in primary neurons after OGD/R. Interestingly, the expression of demethylase ALKBH5 was increased significantly. Overexpression of FTO reduced cleaved caspase-3 levels and inhibited apoptosis, but knockdown of ALKBH5 enhanced cleaved caspase-3 levels and promoted apoptosis. The opposite expression of FTO and ALKBH5 might be that the increased ALKBH5 expression served to compensate for stress responses in the neuronal ischemia/hypoxia. Knockdown of ALKBH5 aggravated neuronal apoptosis, indicating that the compensatory rise of ALKBH5 was likely neuroprotective. The mechanism of how ALKBH5 and FTO co-regulated apoptosis was that the demethylation of ALKBH5 and FTO selectively demethylated the B-cell lymphoma 2 (Bcl-2) transcript, prevented Bcl-2 transcript degradation and induced Bcl-2 protein expression (Xu K. et al., 2020). In addition, Zhang Z. et al. (2020) found that after ischemic stroke, knockdown of YTHDC1 reduced the expression of anti-apoptotic protein Bcl-2 and increased the expression of cleaved caspase-3. Overexpression of YTHDC1 reversed these effects, confirming the protective effect of YTHDC1 on ischemic stroke-induced neuronal apoptosis. Mechanistically, YTHDC1 promoted PTEN mRNA degradation to increase AKT phosphorylation, thus up-regulating the expression of Bcl-2, down-regulating the expression of cleaved caspase-3 and facilitating neuronal survival after ischemic stroke (Zhang Z. et al., 2020).

Researches so far have studied the role of m6A modification on apoptosis in general. However, apoptosis can be divided into two pathways: the mitochondria-dependent pathway (the intrinsic pathway) and the death receptor-dependent pathway (the extrinsic pathway) (Radak et al., 2017). Although the function of m6A modification on mitochondria-dependent pathway has been well documented in CNS injuries, whether the death receptor-dependent pathway is associated with the effects of m6A modification in CNS injuries-induced apoptosis remains unclear and further studies are needed to clarify it.

### Pyroptosis

Pyroptosis is a type of inflammatory PCD that triggered by inflammasomes (Lu et al., 2022). In the process of pyroptosis, inflammasomes activate caspase-1 or caspase-11/4/5, which then cleaves gasdermin D (GSDMD) and separates its N-terminal pore-forming domain (PFD). The oligomers of PFD bind to the cell membrane and form macropores on the membrane, resulting in cell swelling and membrane rupture (Al Mamun et al., 2022). Increasing evidence indicates that pyroptosis is involved in many diseases, including CNS injuries (Mi et al., 2022; Wu et al., 2022).

Since pyroptosis aggravates CNS injuries-caused secondary injury, m6A modification may attenuate brain damage by suppressing pyroptosis. Consistent with this hypothesis, Wang B. et al. (2022) proposed that that in an LPS-induced SBI model, emodin treatment decreased the protein levels of syndecan-1 (SDC-1), NLRP3, caspase-1 and the N-terminal fragment of GSDMD (GSDMD-N) in 1321N1 cells, suggesting that emodin could inhibit pyroptosis. While Nigericin, a NLRP3 activator, reversed the effects of emodin on pyroptosis. Furthermore, emodin promoted m6A levels in NLRP3 by METTL3. METTL3 knockdown reversed the effects of emodin on the mRNA expression and stability of NLRP3, showing that emodin suppressed pyroptosis in SBI by inactivating METTL3-mediated NLRP3 expression (Wang B. et al., 2022). In addition, Diao et al. (2020) found that in cerebral ischemia/reperfusion (I/R) injury, hypothermia down-regulated the expression of pyroptosis-related proteins such as NLRP3, ASC, cleaved caspase-1 and GSDMD p30 in primary hippocampal neurons. Besides, the m6A methylated level of PTEN mRNA was elevated in respond to H/R, whereas this level remained stable after treatment of hypothermia. Up-regulation of the PTEN m6A methylated level alleviated the inhibitory effects of hypothermia on pyroptosis, suggesting that hypothermia protected neurons against H/R-induced pyroptosis *via* m6A-mediated activation of PTEN (Diao et al., 2020).

### Ferroptosis

Ferroptosis is a non-apoptotic form of cell death that depends on iron-mediated lipid free radical formation and accumulation (Peng et al., 2023). It is characterized by increased lipid peroxidation (LPO) leading to cell death through disturbing membrane integrity (Fuhrmann and Brune, 2022). Ferroptosis can be inhibited by glutathione peroxidase 4 (GPX4) and glutathione (GSH), which are key regulators for protecting cells from LPO (Wang M. P. et al., 2022). Change of mitochondrial morphology is a characteristic of ferroptosis, which comprises mitochondrion condensation or swelling, increased membrane density, decreased crista, and ruptured outer membrane (Yang L. et al., 2022).

Ferroptosis contributes to tissue damage in the case of brain injury and inhibition of ferroptosis can provide neuroprotection. Ferroptosis can also be regulated by m6A modification in CNS injuries. Zhang L. et al. (2022) indicated that in intracerebral hemorrhage (ICH) models, silencing of METTL3 relieved Fe^2+^, ROS, LPO, malondialdehyde (MDA) levels, and enhanced GSH levels in oxygen and glucose deprivation/hemin (OGD/H)-treated brain microvascular endothelial cells (BMVECs) and ICH mice, suggesting that METTL3 knockdown inhibited the ferroptosis development in ICH. Furthermore, silencing of METTL3 decreased the m6A levels of GPX4 and increased the mRNA levels of GPX4. GPX4 knockdown neutralized the role of METTL3 on inhibiting ferroptosis in OGD/H-treated BMVECs, demonstrating that METTL3 silencing effectively suppressed ferroptosis by regulating m6A and mRNA levels of GPX4 (Zhang L. et al., 2022).

## Downstream molecules of N6-methyladenosine modification in central nervous system injuries

**FIGURE 2:**
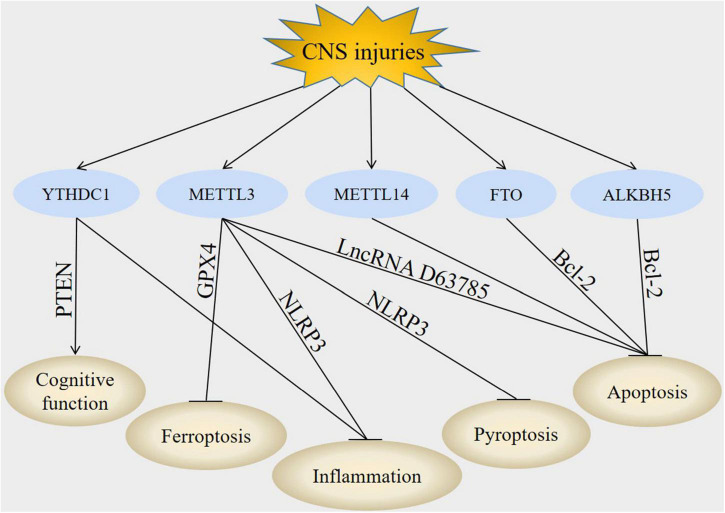
Downstream molecules of m6A modification in CNS injuries. In CNS injuries, regulation of YTHDC1, METTL3, METTL14, FTO, and ALKBH5 led to the modulation of PTEN, GPX4, NLRP3, lncRNA D63785, and Bcl-2. These downstream molecules subsequently improved cognitive function, inhibited inflammation, suppressed apoptosis, decreased pyroptosis and attenuate ferroptosis post-CNS injuries.

The specific mechanisms mediating the functions of m6A modification in CNS injuries have yet to be explained, recent studies have demonstrated that the regulatory factors of m6A modification can target some downstream molecules to play a role in CNS injuries (Figure 2 and Table 2).

**TABLE 2:** The functions and molecular targets of m6A proteins in CNS injuries.

M6A proteins	Models	Animals and/or cells	Beneficial functions of m6A modification	Molecular targets	References
METTL3	ICH SBI Ischemic stroke TBI	Mice, BMVECs Astrocytoma 1321N1 cells SH-SY5Y cells and neurons Mice	Decrease ferroptosis and apoptosis Suppress inflammation and pyroptosis Inhibit cell death and apoptosis Regulate cellular metabolic process	GPX4 NLRP3 LncRNA D63785 /	Wang et al., 2019; Xu S. et al., 2020; Wang B. et al., 2022; Zhang L. et al., 2022
METTL14	TBI	Rats	Reduce apoptosis	/	Yu et al., 2020
FTO	I/R injury TBI	Rats, neurons Mice	Attenuate neuronal damage and apoptosis Repair neurological damage	Bcl-2 /	Xu K. et al., 2020; Yu et al., 2020
ALKBH5	I/R injury	Rats, neurons	Suppress neuronal damage and apoptosis	Bcl-2	Xu K. et al., 2020
YTHDC1	Ischemic stroke	Rats, neurons	Alleviate neurological deficits, increase neuronal survival	PTEN, AKT	Zhang Z. et al., 2020

CNS, central nervous system; ICH, intracerebral hemorrhage; BMVECs, brain microvascular endothelial cells; GPX4, glutathione peroxidase 4; SBI, sepsis brain injury; NLRP3, NLR family pyrin domain containing 3; LncRNA, long non-coding RNA; TBI, traumatic brain injury; I/R, ischemia/reperfusion; Bcl-2, B-cell lymphoma 2; PTEN, phosphatase and tensin homolog; AKT, protein kinase B.

### Phosphatase and tensin homolog

Phosphatase and tensin homolog is encoded by the p ten gene mapped to chromosome 10q23 (Walker et al., 2013). PTEN is a dual-function protein tyrosine phosphatase that dephosphorylates both proteins and lipids. It is also well known for its ability to regulate cell growth and proliferation (Li M. X. et al., 2022). PTEN is thought to exert its effects *via* negative regulation of phosphoinositide 3-kinase (PI3K). Deletion of PTEN firstly activates class I PI3K, then phosphorylates phosphatidylinositol 4,5-biphosphate (PIP2) to phosphatidylinositol 3,4,5-triphosphate (PIP3) and recruits signaling proteins, including AKT and downstream mammalian target of rapamycin (mTOR) activation (Khan et al., 2021; Alcaraz et al., 2022).

Phosphatase and tensin homolog is highly expressed in adult neurons (Gutilla and Steward, 2016). Under pathological conditions such as CNS injuries, PTEN is important for neuronal proliferation, growth and axon regeneration (Fang et al., 2022; Nieuwenhuis and Eva, 2022). M6A modification can also target PTEN to provide neuroprotection. It has been shown that inhibition of m6A-mediated activation of PTEN could protect neurons against neuronal H/R-induced pyroptosis (Diao et al., 2020). Moreover, YTHDC1, a m6A reader, promoted PTEN mRNA degradation to increase AKT phosphorylation, thus facilitating neuronal survival in ischemic stroke (Zhang Z. et al., 2020).

### NLR family pyrin domain containing 3

NLR family pyrin domain containing 3 inflammasome is an intracellular protein complex that has emerged as a key mediator of inflammation in many pathologies (Wang M. et al., 2022). NLRP3 inflammasome includes NLRP3, ASC and protease caspase-1. NLRP3 interacts with ASC after activation and then recruits protease caspase-1 by recognizing pathogen-related molecular patterns (PAMPs) or host-derived danger signal molecules (DAMPs) to promote shear activation. Activated caspase-1 cleaves the precursor IL-1β and IL-18 into mature IL-1β and IL-18, and triggers a series of subsequent inflammatory response (Coll et al., 2022; Wang L. et al., 2022).

The NLRP3 inflammasome is an important defensive component in respond to stress, and its role in CNS injuries has also attracted much attention (Kalra et al., 2022). It has been suggested that inactivation of METTL3-mediated NLRP3 expression could suppress SBI-induced inflammation and pyroptosis in 1321N1 cells (Wang B. et al., 2022).

### B-cell lymphoma 2

B-cell lymphoma 2 is an anti-apoptotic protein encoded by the Bcl-2 gene in the human genome specified as an oncogene, it plays an important role in the mitochondria-mediated intrinsic apoptosis pathway (Flores-Romero et al., 2022; Parrondo et al., 2022). Bcl-2 can bind to the pro-apoptotic proteins BAX/BAK, inhibit their recruitment, thereby blocking the release of cytochrome c and activation of caspases that stimulate apoptosis (Sekar et al., 2022; Xu and Ye, 2022). Abnormal expression of Bcl-2 proteins is a common finding in CNS injuries (Nhu et al., 2021). After brain injury, Bcl-2 promotes cell survival and regulates mitochondrial dynamics like fusion and fission. Thus, activation of Bcl-2 can exert a targeted therapeutic effect and inhibit apoptosis (Wang X. X. et al., 2020).

Bcl-2 could also be regulated by m6A modification in CNS injuries. In a cerebral ischemia-reperfusion injury model, demethylation of ALKBH5 and FTO selectively demethylated Bcl-2 transcript, prevented Bcl-2 transcript degradation and enhanced Bcl-2 protein expression, resulting in decreased apoptosis (Xu K. et al., 2020).

### Glutathione peroxidase 4

Glutathione peroxidase 4, initially called phospholipid hydroperoxide glutathione peroxidase (PHGPX), was first purified in 1982 (Weaver and Skouta, 2022). GPX4 is the main endogenous antioxidant against lipid peroxidation, it also regulates ROS chain reaction caused by iron accumulation (Zhao et al., 2022). GPX4 can reduce complex hydrogen peroxide such as phospholipid hydrogen peroxide and cholesterol hydrogen peroxide to their respective peroxidation products, thus blocking the chain reaction of LPO and suppressing ferroptosis (Wang Y. et al., 2022). The functional activity of GPX4 depends on the biosynthesis of tripeptide GSH, depletion of GSH causes GPX4 inactivation and increases intracellular LPO (Li D. et al., 2022). Recently, a number of GPX4-targeted therapeutic regimens have been proposed for the treatment of CNS injuries (Huang et al., 2022; Peeples and Genaro-Mattos, 2022; Yuan et al., 2022).

The effects of m6A modification on GPX4 have been reported. It has been shown that knockdown of METTL3 inhibited ferroptosis, decreased the m6A levels of GPX4 and increased the mRNA levels of GPX4 in OGD/H-treated BMVECs and ICH mice. GPX4 knockdown reversed the role of METTL3 on relieving Fe^2+^, ROS, LPO and MDA levels in OGD/H-treated BMVECs and ICH mice, implying that METTL3 silencing suppressed ferroptosis by regulating m6A and mRNA levels of GPX4 in ICH (Zhang L. et al., 2022).

### Long non-coding RNA D63785

Long non-coding RNA refer to the transcripts of non-coding RNAs that are >200 nucleotides in length, they include five different subtypes: sense lncRNA, antisense lncRNA, bidirectional lncRNA, intergenic lncRNA and intronic lncRNA (Cao et al., 2022). LncRNAs were primarily considered as simply transcriptional by-products, recent research have found that they regulate various physiological and pathophysiological processes, such as immunity, cell differentiation and proliferation (Zhang and Wang, 2019). LncRNAs regulate gene expression at the epigenetic, transcriptional, post-transcriptional and chromatin remodeling levels *via* interacting with the 3′ untranslated region (UTR) of mRNA (Chen et al., 2021). LncRNAs also interact with other biomolecules including deoxyribonucleic acids (DNAs), RNAs and proteins through several mechanisms, including acting as inhibitory sponges for miRNAs, participating in chromatin remodeling and affecting protein stability (Wang J. et al., 2022).

The crosstalk between m6A modification and lncRNA has been explained in ischemic stroke. In SH-SY5Y cells and primary murine neurons, OGD/R induced cell death and apoptosis through activation of METTL3-dependent lncRNA D63785 m6A methylation (Xu S. et al., 2020).

## Crosstalk between N6-methyladenosine modification and RNAs in central nervous system injuries

Although the relationships between m6A modification and RNAs has been found in CNS injuries, the detailed mechanisms of how m6A modification regulated RNAs were not explained in CNS injuries. However, in other models, accumulating evidences have identified that m6A modification had regulatory effects on RNAs, including their splicing, processing, translation and degradation.

### N6-methyladenosine modification modulates the splicing and maturation of RNAs

Nascent transcripts synthesized from DNA must undergo splicing before transformation into mature transcripts with biological functions and m6A modification regulates gene expression by interfering with this process. It has been shown that HNRNPC, HNRNPG, and HNRNPA2B1 were associated with mRNA structure switching, thus regulating gene expression (Liu et al., 2015). M6A modification could affect the binding between HNRNPC and RNA, therefore regulating the alternative splicing and processing of target RNA, resulting in RNA maturation (Liu et al., 2015). Moreover, FTO has been found in the nucleoplasm in a speckle-like manner and partially co-localized with splicing or splicing-associated speckle factors (Jia et al., 2011). FTO-regulated m6A modification was enriched in exon regions on both sides of the 5′ and 3′ splicing sites. These regions overlapped spatially with the enhancer-binding region of the serine- and arginine-rich splicing factor (SRSF), which modulated mRNA splicing (Zhao et al., 2014). It has been suggested that depletion of FTO enhanced the m6A level and promoted the affinity of SRSF2 binding with RNA, thereby increasing the number of target exons and inducing RNA maturation (Zhu et al., 2021). Furthermore, the m6A reader YTHDC1 could recruit the splicing factor SRSF3 to promote exon inclusion but antagonize SRSF10 mRNA binding, which facilitated exon skipping (Xiao et al., 2016). In addition, transactivation responsive RNA-binding protein 2 (TARBP2) recruited the MTCs to deposit m6A marks on transcripts, leading to intron retention *via* the splicing factor SRSF1 (Fish et al., 2019). Besides, METTL3 and YTHDC1 could modulate the maturation of circRNA ZNF609, both METTL3 and YTHDC1 displayed specific m6A signatures that controlled the accumulation of circRNA ZNF609 (Di Timoteo et al., 2020). These results strongly confirmed the essential role of m6A RNA modification in RNA splicing and maturation.

### N6-methyladenosine modification regulates the translation of RNAs

The mechanism by which m6A modification improves the translation efficiency is mainly dependent on the binding of reader proteins to protein factors that are required in the translation process, the presence of m6A in exons and surrounding stop codon regions may have an impact on protein production (Liu R. et al., 2022). It has been indicated that ablation of METTL3 enhanced translation efficiency in mouse embryonic stem cells (mESCs) and embryoid bodies (EBs), demonstrating that m6A played a translational regulatory role (Geula et al., 2015). Besides, Lin et al. (2019) found that during the epithelial-mesenchymal transition, YTHDF1 mediates the coding sequence (CDS) m6A-enhanced translation elongation of Snail mRNA *via* interactions with the translation elongation factor eEF2. Moreover, Liu et al. (2020) showed that the eukaryotic translation initiation factor 3 subunit C (EIF3C) was a direct target of YTHDF1. By binding to m6A-modified EIF3C mRNA, YTHDF1 enhanced the translation of EIF3C in an m6A-dependent manner and promoted overall translation output. Furthermore, Jin et al. (2019) found that METTL3, YTHDF1/3 and eIF3b directly promoted the translation of YAP mRNA through interaction with the translation initiation process. In addition, Yang et al. (2017) identified that m6A residues were abundant in circRNAs and could drive efficient initiation of protein translation from circRNAs. The m6A-induced translation of circRNAs could be increased by METTL3/14 and decreased by FTO (Yang et al., 2017). Besides, Di Timoteo et al. (2020) discovered that circRNA ZNF609 translation was modulated through recognition by YTHDF3 and eIF4G2.

### N6-methyladenosine modification controls the stability and degradation of RNAs

The stability and degradation of RNAs are critical for the response of living organisms to changeable environments and m6A-containing transcripts can mediate RNA stability and degradation *via* different molecular mechanisms. Wang Y. et al. (2014) indicated that knockdown of METTL3/14 increased the stability of target mRNAs and prevented mRNA degradation in mouse embryonic stem cells, suggesting that mRNA instability was associated with m6A RNA modification. Moreover, Cai et al. (2021) reported that IGF2BP1 increased the stability of YES1 mRNA and prevented its degradation. Furthermore, Wang X. et al. (2014) showed that YTHDF2 selectively recognized an m6A site according to the carboxyl-terminal domain, and the amino-terminal domain was responsible for translocating the YTHDF2-mRNA complex to a cellular RNA decay site to regulate mRNA degradation. In addition, Wu et al. (2021) suggested that METTL3-mediated m6A modification stabilized the expression of circRNA CUX1, which conferred radio-resistance in hypopharyngeal squamous cell carcinoma. Collectively, these data revealed the role of m6A RNA modification in RNAs stability and degradation.

## Possible research directions of N6-methyladenosine modification in central nervous system injuries

Central nervous system injuries, caused by cerebrovascular pathologies or mechanical contusions, comprise a diverse group of pathological processes, including autophagy, oxidative stress, inflammation and apoptosis (Nakamura et al., 2020). Although the functions of m6A modification on CNS injuries-induced neurological impairment, inflammation, apoptosis, pyroptosis and ferroptosis have been widely described, its roles in autophagy, oxidative stress, axonal and synaptic regeneration have not been illustrated.

### Autophagy

Autophagy is a highly conserved intracellular clearance mechanism that functions to maintain cellular homeostasis by engulfing cellular targets, including damaged organelles, unfolded proteins and pathogens (Liao M. F. et al., 2022). When autophagy is activated, the damaged organelles are enclosed by an isolation membrane to form autophagosome (Zhang et al., 2021a). Autophagosome then fuses with lysosome to form autolysosome and the damaged organelles are degraded by lysosomal enzymes (Zhang and Wang, 2018a).

The functions of m6A modification in autophagy have also been well established. It has been shown that Unc-51-like kinase 1 (ULK1) expression was regulated in an FTO-m6A-dependent and YTHDF2-mediated manner in gastric cancer. Knockdown of FTO reversed cisplatin resistance of gastric cancer cells both *in vitro* and *in vivo*, which was attributed to the inhibition of ULK1-mediated autophagy (Zhang Y. et al., 2022). Furthermore, in diabetic skin models, knockdown of endogenous YTHDC1 resulted in a blockade of autophagic flux and delayed wound healing by droving SQSTM1 mRNA degradation in the nucleus (Liang et al., 2022). Therefore, m6A modification may also intervene autophagy in CNS injuries. However, further studies are needed to verify it.

### Oxidative stress

Oxidative stress, defined as imbalance between the biological systems leading to the generation of oxidant (free) radicals and the systems responsible for the removal of free radicals, is harmful to cells due to the excessive generation of oxidant compounds such as ROS and reactive nitrogen species (RNS) (Zhang and Wang, 2018b). Excessive generation of ROS and RNS due to depletion of the antioxidant system or excitotoxicity leads to the oxidation of biological molecules such as lipids, proteins and DNA, resulting in oxidative damage in cells, tissues and organs (Khatri et al., 2018).

There were also researches suggesting that m6A modification could regulate oxidative stress. Ding et al. (2022) showed that in bovine granulosa cells, FTO and YTHDF2 regulated MAX network transcriptional repressor (MNT) expression through m6A modification. FTO overexpression alleviated cadmium (Cd)-induced oxidative stress as proven by increased expression of nuclear factor erythroid 2-related factor-2 (Nrf2), superoxide dismutase (SOD), catalase (CAT) and NAD(P)H: quinone oxidoreductase 1 (NQO1), and reduced expression of MDA. However, this process could be reversed using si-MNT. Moreover, Zhuang et al. (2019) found that in clear cell renal cell carcinoma (ccRCC), FTO induced oxidative stress and increased reactive oxygen (ROS) levels by reducing m6A methylation of peroxisome proliferator-activated receptor gamma coactivator-1 α (PGC1α) and increasing PGC1α mRNA translation efficiency. Therefore, the role of m6A modification in CNS injuries induced-oxidative stress needed to be further studied.

### Axonal and synaptic regeneration

Central nervous system injuries lead to a rapid loss of neurons and axons which accounts for the loss of nerve connections during the acute phase and subsequently induces various degrees of plasticity during the spontaneous recovery phase (Cramer, 2018). It has been shown that stroke-mediated injury could induce axon sprouting, dendritic branching and synaptogenesis for remapping of neural circuits (Carmichael et al., 2017). The speed of axonal and synaptic regeneration is affected by axonal transportation and neurotrophic growth factors, which are secreted by Schwann cells (SCs). In respond to CNS injuries, SCs are activated and involved in the entire process of injury and regeneration. The proliferating SCs form Bungner bands, which guide the growth of newly sprouting axons (Chu et al., 2022).

After nerve injury, m6A modification may have the function of regulating axonal regeneration and synaptic regeneration. Zhang et al. (2021b) performed MeRIP-seq to reveal the m6A methylation landscape in peripheral nervous injury (PNI). They found that 4,014 m6A peaks were significantly altered after PNI. Moreover, GO analysis and KEGG pathway analysis showed that these genes were mainly involved in axon regeneration (Zhang et al., 2021b). Furthermore, Wang X. L. et al. (2022) reported that in a mouse neuropathic pain (NP) model, downregulation of FTO in the anterior cingulate cortex (ACC) could promote angiogenesis, axon growth and neural plasticity as proven by up-regulation of matrix metalloproteinase-9 (MMP-9), decreased levels of precursor brain-derived neurotrophic factor (proBDNF) and increased levels of mature brain-derived neurotrophic factor (mBDNF). The current researches have focused m6A modification on the peripheral nerve injury, which had certain reference for the application of m6A modification in the CNS injuries.

## Concluding remarks

N6-methyladenosine modification plays essential roles in CNS injuries and participates in a number of cellular and molecular processes of CNS injuries. In this review, we summarize the abnormal expression of m6A modification proteins, the function of m6A modification and the crosstalk between m6A modification and RNAs in CNS injuries. These observations make m6A modification to be attractive therapeutic targets for patients suffering from CNS injuries. Moreover, microarray, proteomic and metabolomic analyses of the downstream moleculars of m6A modification may offer new avenues for restoring normal neuronal network and blocking the vital nodes promoting brain damage. Continued discoveries in this field will bring novel insights on m6A modification involved in biological functions and disease progression. Ultimately, m6A modification may hold promise for clinical challenges.

## Author contributions

LZ conceived the whole work design, finished the figures and tables, and played a vital role in manuscript submissions. MT finished the original manuscript. LM revised the manuscript. All authors contributed to the article and approved the submitted version.
